# Pharmacological inhibition of *I*_K__1_ by PA‐6 in isolated rat hearts affects ventricular repolarization and refractoriness

**DOI:** 10.14814/phy2.12734

**Published:** 2016-04-25

**Authors:** Mark A. Skarsfeldt, Helena Carstensen, Lasse Skibsbye, Chuyi Tang, Rikke Buhl, Bo H. Bentzen, Thomas Jespersen

**Affiliations:** ^1^ Department of Biomedical SciencesFaculty of Health and Medical SciencesUniversity of CopenhagenCopenhagenDenmark; ^2^ Department of Large Animal SciencesFaculty of Health and Medical SciencesUniversity of CopenhagenCopenhagenDenmark

**Keywords:** Action potential, *I*_K__1_, inward rectifier current, Kir2.x channels, pentamidine

## Abstract

The inwardly rectifying potassium current (*I*_K__1_) conducted through K_ir_2.X channels contribute to repolarization of the cardiac action potential and to stabilization of the resting membrane potential in cardiomyocytes. Our aim was to investigate the effect of the recently discovered *I*_K__1_ inhibitor PA‐6 on action potential repolarization and refractoriness in isolated rat hearts. Transiently transfected HEK‐293 cells expressing *I*_K__1_ were voltage‐clamped with ramp protocols. Langendorff‐perfused heart experiments were performed on male Sprague–Dawley rats, effective refractory period, Wenckebach cycle length, and ventricular effective refractory period were determined following 200 nmol/L PA‐6 perfusion. 200 nmol/L PA‐6 resulted in a significant time‐latency in drug effect on the *I*_K__1_ current expressed in HEK‐293 cells, giving rise to a maximal effect at 20 min. In the Langendorff‐perfused heart experiments, PA‐6 prolonged the ventricular action potential duration at 90% repolarization (from 41.8 ± 6.5 msec to 72.6 ± 21.1 msec, 74% compared to baseline, *P* < 0.01, *n* = 6). In parallel, PA‐6 significantly prolonged the ventricular effective refractory period compared to baseline (from 34.8 ± 4.6 msec to 58.1 ± 14.7 msec, 67%, *P* < 0.01, *n* = 6). PA‐6 increased the short‐term beat‐to‐beat variability and ventricular fibrillation was observed in two of six hearts. Neither atrial ERP nor duration of atrial fibrillation was altered following PA‐6 application. The results show that pharmacological inhibition of cardiac *I*_K__1_ affects ventricular action potential repolarization and refractoriness and increases the risk of ventricular arrhythmia in isolated rat hearts.

## Introduction

The inward rectifier potassium current (*I*_K1_) contributes to repolarization in cardiomyocytes (Dhamoon and Jalife 2005). It is important for setting the diastolic membrane potential (Phase 4 of the cardiac action potential, also named resting membrane potential), and for the late repolarization of the cardiac action potential (Phase 3) where it constitutes a part of the repolarization reserve currents (Ibarra et al. 1991). Consequently, changes in *I*_K1_ have significant effects on the cardiac action potential morphology, the excitability of the heart and thereby possibly contribute to or protect against cardiac arrhythmia (Schmitt et al. 2014).

*I*_K1_ is an inwardly rectifying potassium current preferring inward over outward conductance due to block of the pore at depolarized membrane potentials by intracellular divalent cations such as Mg^2+^ and Ca^2+^ and by polyamines such as spermine and spermidine (Anumonwo and Lopatin 2010). There are four members of this family (K_ir_2.1‐K_ir_2.4), and in cardiac tissue K_ir_2.1 is the predominant expressed protein. Loss of function mutations in *KCNJ2,* which encodes K_ir_2.1, is reported in Andersen–Tawil syndrome (Plaster et al. 2001) and gain of function can cause shortening of the QT‐interval, both of which increases the risk of ventricular arrhythmias (Anumonwo and Lopatin 2010). However, the exact role of *I*_K1_ in cardiac arrhythmias is poorly understood, mainly due to lack of specific and efficacious *I*_K1_ modulators.

In the past, *I*_K1_ has been blocked by nonspecific compounds like RP‐58866 (Yang et al. 1999), MS‐551 (Sen et al. 1998), chloroquine (Rodriguez‐Menchaca et al. 2008), tamoxifen (Ponce‐Balbuena et al. 2009), or by cations such as barium and cesium (Hibino et al. 2010)(reviewed by [van der Heyden and Jespersen 2016]). The cations were originally used for characterizing the *I*_K1_ current and were followed by more potent and specific molecules that eased the characterization of *I*_K1_ in vitro and in vivo. However, all of the mentioned compounds affect other targets in addition to the K_ir_2.x currents. More recently a probe report showed potent block with high selectivity toward K_ir_2.x using the compound, ML133 (Wu et al. 2010).

Cardiac arrhythmias associated with QTc prolongation and U‐wave alternations have been observed when pentamidine is used for treatment of protozoal infections. As U‐wave deflections on the ECG (Electrocardiogram) are associated with *I*_K1_, De Boer et al., (de Boer et al. 2010b) investigated the mechanism and action of pentamidine mediated *I*_K1_ block. They concluded that pentamidine inhibits *I*_K1_ in isolated adult canine ventricular cardiomyocytes and that pentamidine blocks the pore region of K_ir_2.1 from the cytoplasmic side (de Boer et al. 2010b). To obtain a more specific and potent *I*_K1_ block, Takanari et al., examined several analogs of pentamidine, and found PA‐6 to show the highest affinity for cardiac *I*_K1_. PA‐6 blocks human K_ir_2.1 and 2.2 and mouse K_ir_ 2.1, 2.2, and 2.3 in the range 12–15 nmol/L (Takanari et al. 2013). PA‐6 thereby has a more than 10‐fold lower IC_50_ value for *I*_K1_ block than ML133 (180 nmol/L) (Takanari et al. 2013) (Wu et al. 2010). Furthermore, PA‐6 did not significantly affect *I*_Nav_, *I*_Ca‐L_, *I*_To_, *I*_Kr_, and *I*_Ks_ at 200 nmol/L (Takanari et al. 2013). In the same study, current clamping of isolated canine ventricular cardiomyocytes revealed a drastic prolonged action potential duration and short‐term variability following PA‐6 application, confirming the prominent role of *I*_K1_ in repolarization (Takanari et al. 2013). So far, the role of cardiac *I*_K1_ has been investigated using barium chloride, however, Ba^2+^ is not a specific blocker of *I*_K1_, as it also blocks other inwardly K^+^ rectifiers (Lesage et al. 1995). Furthermore, Ba^2+^ is toxic and not tolerable in vivo (de Boer et al. 2010a). In order to investigate how cardiac electrophysiology is affected by a specific *I*_K1_ block we here use the *I*_K1_ inhibitor PA‐6 to examine the electrophysiological effects of *I*_K1_ inhibition in Langendorff‐perfused rat hearts.

## Methods

### Whole‐cell patch‐clamp recordings

#### Cell line preparation

*I*_K1_ currents were investigated in transiently transfected HEK‐293 cells, which were grown in Dulbecco's modified Eagle's medium (DMEM; Life Technology, NY) supplemented with 10% fetal bovine serum (FBS; Sigma‐Aldrich, St. Louis) and 1% streptomycin (Invitrogen, Naerum, Denmark) at 37°C in a 5% CO_2_ atmosphere.

#### Molecular cloning and transfection

Complementary DNAs encoding human K_ir_2.1 (GenBank ACC. NM_000891) were subcloned into the pXOOM vector, as previously described in (Yang et al. 2010). To reconstitute *I*_K1_ currents HEK‐293 cells were transiently transfected with 1 *μ*g pXOOM‐hK_ir_2.1 and 0.2 *μ*g pcDNA3‐eGFP (reporter gene). Transfections were performed using Lipofectamine 2000 (Invitrogen, Naerum, Denmark) according to the manufacturer's instructions. The cells were used for patch‐clamp recordings 36–48 h after transfection.

#### Solutions and chemicals

The following physiological extracellular solution was used for the electrophysiological experiments, containing (in mmol/L): NaCl 140, KCl 4, CaCl_2_ 2, MgCl_2_ 1, HEPES 10, D‐Glucose 10 (pH 7.4 with NaOH). The pipette solution consisted of (in mmol/L) KCl 140, Na_2_ATP 1, EGTA 2, HEPES 10, CaCl_2_ 0.1, MgCl_2_ 1, D‐Glucose 10 (pH 7.4 with KOH).

#### Electrophysiological methods

Patch‐clamp recordings were performed as previously described (Grunnet et al. 2001) (Yuan et al. 2014). HEK‐293 cells were voltage‐clamped with ramp protocols. Whole‐cell currents were measured at room temperature (20–22°C) with an EPC‐9 amplifier and Pulse software (both from HEKA Elektronik, Lambrecht, Germany). Borosilicate glass pipettes were pulled on a DPZ‐Universal puller (Zeitz Instrumente, Munich, Germany). The pipettes had a resistance of 1.5–2.5 MΩ. The series resistances for whole‐cell configuration were 2–5 MΩ and were 80% compensated. At least 1.0 GΩ were achieved in all experiments.

### Isolated Langendorff‐perfused rat heart preparations

The experiments followed the European Community Guidelines for the Care and Use of Experimental Animals and were performed under License No. 2010/561‐1897. All procedures performed involving animals were in accordance with the ethical standards of the institution at which the studies were conducted and apply to Danish legislations. This article does not contain any studies with human participants.

The Langendorff‐perfused heart experiments were performed on male Sprague–Dawley rats (Taconic A/S, Denmark). On the day of the experiment the rats were weighed (BW 300–400 g) and anesthetized with a subcutaneous injection of fentanyl‐midazolam mixture, 5 mg/mL, dose 0.3 mL/100 g BW.

The rats were ventilated (4 mL/60 strokes/min) through a rodent ventilator (7025 Rodent ventilator, Ugo Basile, Italy). Tracheostomies were performed and the hearts were excised and cannulated through a small puncture of the aorta, near the aortic arch, and connected to the Langendorff retrograde perfusion setup (Hugo Sachs Elektronik, Harvard Apparatus GmbH, Germany). The hearts were retrogradely perfused at a constant perfusion pressure of 80 mm Hg with a 37°C, pH 7.4, Krebs–Henseleit buffer (NaCl 120.0, NaHCO_3_ 25.0, KCl 4.0, MgSO_4_ 0.6, NaH_2_PO_4_ 0.6, CaCl_2_ 2.5, Glucose 11.0, all in mmol/L) saturated with 95% O_2_ and 5% CO_2_. The aortic perfusion pressure was determined with an ISOTEC transducer (Hugo Sachs Elektronik, Harvard Apparatus GmbH, Germany) and the coronary flow was measured with an ultrasonic flowmeter (Transonic Systems INC, USA). Both were connected to an amplifier (Hugo Sachs Elektronik, Harvard Apparatus GmbH, Germany). The electrical activity of the rat hearts were measured by using volume conducted electrocardiograms (ECGs) and by placing two epicardial monophasic action potential (MAP) electrodes on the right ventricle. The hearts were immersed into a temperature‐controlled and carbonated bath containing Krebs–Henseleit buffer. Perfusion pressure, coronary flow, ECG and MAP analog signals were sampled at a frequency of 1k/s and were converted by a 16/30 data acquisition system from PowerLab systems (ADInstruments, UK.) and monitored by using LabChart 7 software (ADInstruments, UK.)

The hearts were left to stabilize for 30 min at intrinsic heart rhythm. A bipolar pacing electrode was placed on the right atrial appendage and a second pacing electrode was positioned on the right ventricle. Epicardial pacing‐stimulation was applied using square pulses of 2 msec durations at twice diastolic threshold at 150 basic cycle lengths (BCL). Right atrial and right ventricular effective refractory periods (ERPs) were measured by application of 10 (S_1_) regular pulses followed by a premature extra stimulus (S_2_) with the time between the S1 and the S2 stimuli increased by 2 msec increments until the refractory period was identified. Furthermore, the Wenckebach cycle length (WCL) was determined by pacing with decreasing cycle lengths until a 2:1 AV block appeared.

After the initial 30 min stabilization period atrial ERP (aERP), WCL, and ventricular ERP (vERP) were determined and the heart preparations were randomized into treatment with either 200 nmol/L PA‐6 to the drug‐treated group or equivalent amount of DMSO to the time‐matched control (named DMSO) group. The same parameters were determined every 15 min over a 90 min drug infusion period.

### Compound

The pentamidine analog PA‐6 (C31H32N4O2) was synthesized by Syngene, Bangalore, India. The compound was dissolved in DMSO and the DMSO concentration in the experiments was 0.002%. The compound was added from a 10 mmol/L stock to the Krebs–Henseleit solution used in the experiments yielding a final concentration of 200 nmol/L.

### Data analysis

Whole‐cell patch‐clamp data were analyzed using IGOR Pro (WaveMetrics, Lake Oswego) and GraphPad Prism software (GraphPad Software, San Diego). Data are presented as mean ± SEM.

All Langendorff data were analyzed using Labchart 7 (ADInstruments, UK) and figures were processed in GraphPad Prism (GraphPad Software, USA).

Data represent the mean ± standard error of mean. Two‐tailed paired t‐tests were used to compare baseline with the effect of 90 min perfusion of 200 nmol/L PA‐6 on aERP, vERP, WCL, and action potential duration at 90% repolarization (APD_90_). The APD_90_s were a mean of 40 monophasic action potentials.*P < 0.05 was considered significant. **P < 0.01, ***P < 0.0001. The heart rate variability was evaluated using a Poincare plot. Consecutive R–R intervals from the right ventricle were plotted as 40 *n* + 1 from ventricular maps. The instability of the beat‐to‐beat heart rate was characterized by short‐term variability (STV) and was calculated from 40 subsequent APD_90_s using (STV = $\sum |D_{n+1}-D_{n}|/\left[40\times \sqrt{2}\right]$).

## Results

### Time‐dependent PA‐6 inhibition of *I*_K1_

**Figure 1:**
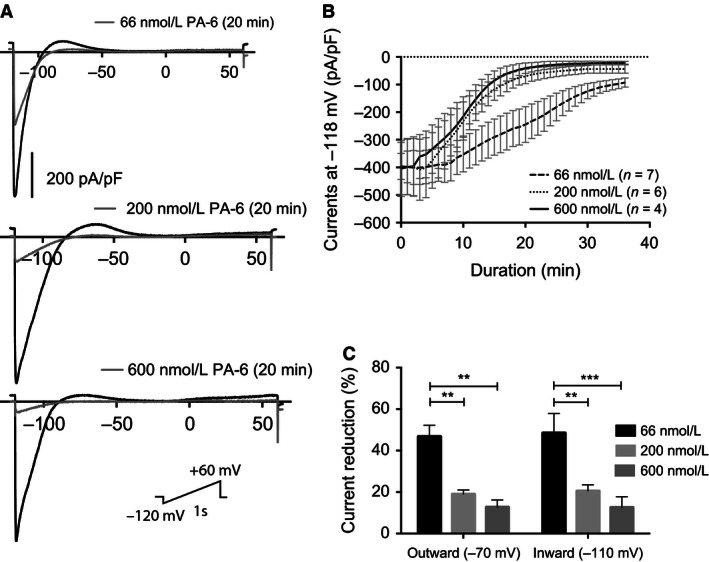
Whole‐cell patch clamping of HEK‐293 cells expressing *I*_K__1_. (A) Representative traces. Currents were recorded in response to a 1000 msec voltage ramp protocol from −120 to +60 mV from a holding potential of −79 mV. 600 nmol/L or 200 nmol/L or 66 nmol/L of PA‐6 were added to the perfusate after getting stable whole‐cell patch‐clamp current. *I*_K__1_ currents were following blocked with different levels by PA‐6 (gray traces). (B) Current‐time relationships of *I*_K__1_ during PA‐6 blockage. Normalized current density with average ± SEM values measured at −118 mV (currents were elicited by the ramp protocol described above). (C) Outward and inward currents measured at −70 mV and −110 mV, respectively, 20 min after application of PA‐6 at 66 nmol/L, 200 nmol/L and 600 nmol/L. 2 way ANOVA followed by Dunnett′s MC test, error bars represent mean ± SEM, *P < 0.05 was considered significant. **P < 0.01, ***P < 0.0001.

Because the PA‐6 binding site on the K_ir_2.1 channel is located at the cytosolic part of the channel, the onset of current inhibition was slow when the drug was applied to the extracellular solution, as also observed by De Boer et al. (de Boer et al. 2010b) and Takanari et al. (Takanari et al. 2013). To obtain a clearer picture of the time‐dependence of *I*_K1_ blockage voltage clamp experiments on HEK‐293/hKir2.1 transiently transfected cells were performed (Fig. 1A). A significant time‐latency in drug effect was observed, giving rise to a maximal effect at 20 min after application of 200 and 600 nmol/L PA‐6. Application of 66 nmol/L reached a maximal inhibitory effect of 75.8% after 35 min of drug application (Fig. 1B). To address the relative inhibition of the inward and outward potassium current following PA‐6 inhibition current reduction was measured at −70 mV (outward K^+^ current) and −110 mV (inward K^+^ current) (Fig. 1C). The K^+^ reversal potential was found to be approximately – 90 mV. At the three PA‐6 concentrations investigated a similar block of outward and inward *I*_K1_ was found.

### Electrophysiological investigations on Langendorff‐perfused rat hearts

To investigate the electrophysiological effects of PA‐6 on the heart, in a system that allows the free concentration of PA‐6 to be precisely controlled, hearts from adult male Sprague–Dawley rats were removed and mounted in a Langendorff set‐up. Here, retrograde perfusion through the aorta of oxygenated saline buffer ensures the viability of the heart for hours and using electrical stimulation it is possible to pace and quantitate the refractoriness of both atria and ventricle. The hearts were paced at a basic cycle length of 150 msec to overrule intrinsic beating and the electrical activity was measured with both monophasic action potential (MAP) and ECG electrodes.

**Figure 2:**
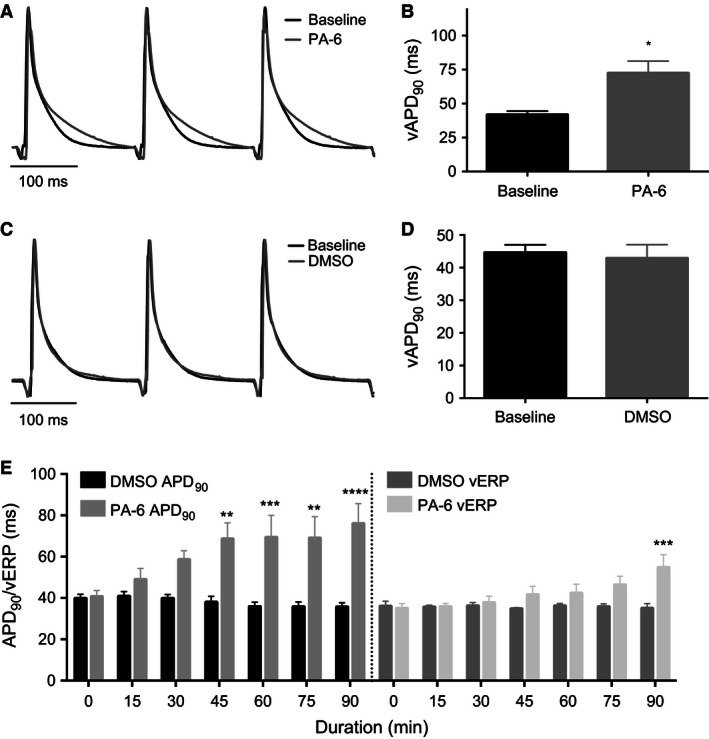
Effect of PA‐6 on ventricular APD_90_. (A) Representative MAP recordings before and after 200 nmol/L PA‐6 application. (B) Summary of vAPD_90_ values before and after application of 200 nmol/L PA‐6 at 90 min. Individual data displayed in Figure 3G. Baseline mean 41.8 ± 6.5 msec, PA‐6 application increased to 72.6 ± 21.1 msec, percent‐increase 74% compared to baseline, *P* < 0.05, *n* = 6) two‐tailed *t*‐test, error bars represent mean ± SEM. (C) Representative MAP recordings before and after application of DMSO. (D) Summary of vAPD_90_ values before and after application of DMSO at 90 min. Individual data displayed in Figure 3C. Baseline mean 44.6 ± 5.3 msec, DMSO application decreased to 42.9 ± 9.3 msec, *n* = 5 two‐tailed *t*‐test, error bars represent mean ± SEM. (E) Time‐dependent change in ventricular APD_90_ and ERP following PA‐6 application. 2‐way ANOVA followed by Bonferroni′s MC post test, error bars represent mean ± SEM, *n* = 5, *P < 0.05 was considered significant. **P < 0.01, ***P < 0.0001.

**Figure 3:**
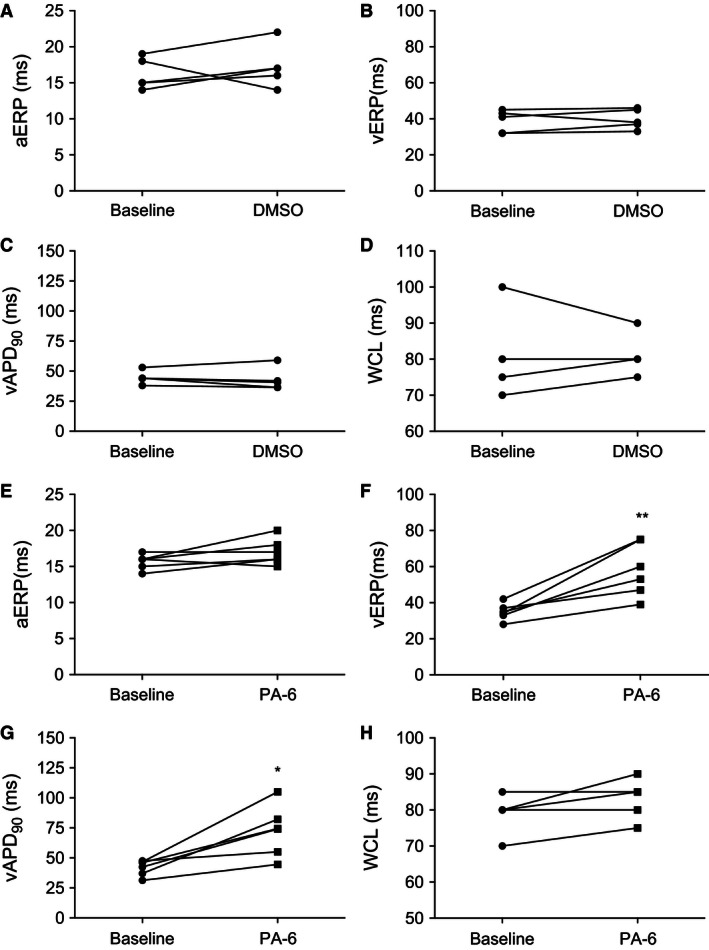
Individual effects of PA‐6. (A, E) Effect of either DMSO or 200 nmol/L PA‐6 on rat aERP, (B, F) vERP, (C, G) vAPD_90_ and (D, H) WCL. Two‐tailed paired *t*‐tests were used to compare baseline with the effect of 90 min perfusion of 200 nmol/L PA‐6 on aERP, vERP, vAPD_90_ and WCL. aERP (*n* = 6, ns, *P* = 0.12); vERP (*n* = 6, *P* = 0.0059); vAPD_90_ (*n* = 6, *P* = 0.0109); WCL (*n* = 5, ns, *P* = 0.09939), *P < 0.05 was considered significant. **P < 0.01, ***P < 0.0001.

Due to the time latency in drug effect on *I*_K1_ PA‐6 was applied to the perfusion buffer for 90 min (Fig. 2A,B). A time‐matched control (DMSO) group was included to investigate time stability of the experimental procedure (Fig. 2C,D). After 90 min perfusion with 200 nmol/L PA‐6 the morphology of the ventricular MAPs was drastically changed showing a prolongation of repolarization in the later part of the action potential, (APD_90_ from 41.8 ± 6.5 msec to 72.6 ± 21.1 msec, 74% compared to baseline, *P* < 0.01, *n* = 6) (Figs. 2A,B and 3G). In the DMSO group neither a change in action potential morphology nor in action potential duration at 90% repolarization (APD_90_) was observed (Figs. 2C,D, and 3C). The time‐dependency of PA‐6 effect were analyzed each 15 min. Significant prolongation of vAPD_90_ was observed after 45 min (from 41.8 ± 6.5 msec to 71.45 ± 16.9 msec), 71% compared to baseline, *P* < 0.05, *n* = 6) (Fig. 2E) while significant ventricular effective refractory period (vERP) prolongation was found 90 min after application of 200 nmol/L PA‐6 (from 34.8 ± 4.6 msec to 58.1 ± 14.7 msec, 67%, *P* < 0.01, *n* = 6) (Figs. 2E and 3F). Interestingly, the atrial ERP was not affected by PA‐6 when compared with baseline (*P* = 0.12, *n* = 6) (Fig. 3E) and neither was the Wenckebach cycle length (WCL), which is a measure of AV‐nodal refractoriness, altered by PA‐6 compared to baseline (*P* = 0.09, *n* = 6) (Fig. 3H). Neither of these parameters where changed in time‐matched controls (Fig. 3A–D).

### Effect of PA‐6 on electrical stability

**Figure 4:**
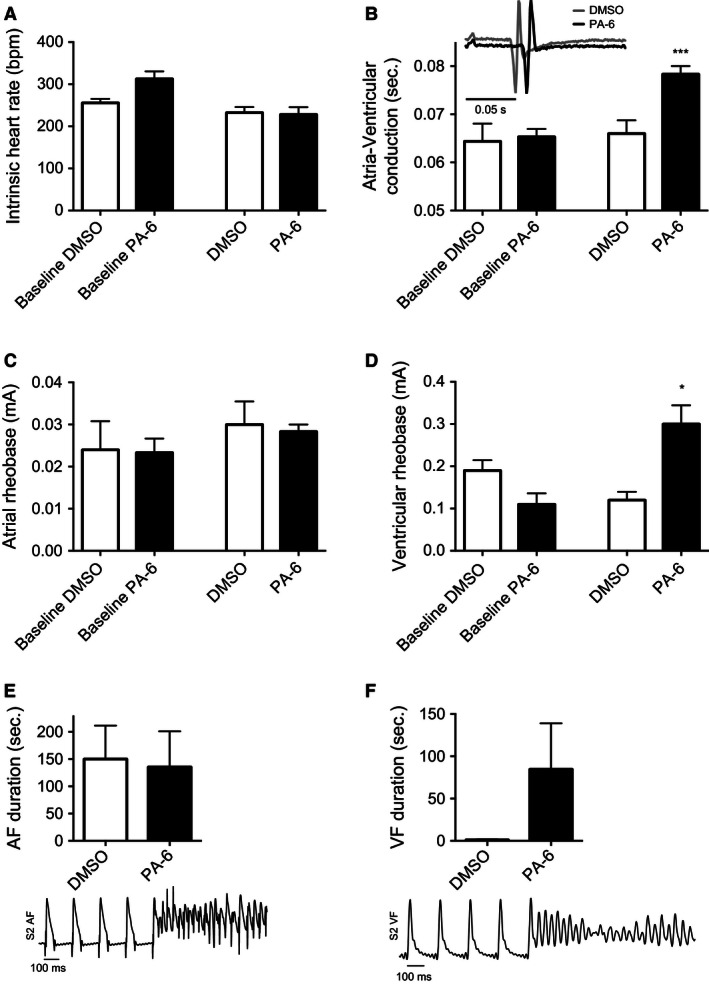
Heart rate, conduction time, atrial and ventricular rheobase, AF and VF duration. (A) Intrinsic heart rate before and after administration of DMSO or PA‐6. DMSO or PA‐6 did not significantly affect the intrinsic heart rate. (B) Atria‐Ventricular conduction with representative ECG. DMSO did not prolong the conduction time between the atrias and ventricle, but PA‐6 increased the conduction time with 0.013 sec, *P* < 0.001. Insert, illustration of ECG following 90 min application of DMSO or PA‐6. (C) Atrial rheobase, DMSO or PA‐6 did not increase the rheobase of the atria. (D) Ventricular rheobase, the ventricular rheobase was significantly increased with 173% in the ventricles during application of PA‐6, *P* = 0.014, *n* = 6. (E) S2‐stimuli‐induced AF. No difference between baseline and DMSO or PA‐6 was observed, *n* = 6, two‐tailed *t*‐test, error bars represent mean ± SEM. (F) S2‐induced VF duration. No significant difference was observed when comparing baseline and DMSO in two‐tailed *t*‐test, *n* = 6. PA‐6 the VF duration increased but was not significant, *P* = 0.18, two‐tailed *t*‐test, error bars represent mean ± SEM, *P < 0.05 was considered significant. **P < 0.01, ***P < 0.0001.

The effect of PA‐6 on beating frequency, conduction and action potential activation current (rheobase) was analyzed prior to PA‐6 and DMSO application (baseline) and after 90 min of application (Fig. 4A–D). PA‐6 did not have an effect on intrinsic heart rate (Fig. 4A), but did reduce atria‐ventricular conduction time (Fig. 4B), measured from the start of the P‐wave to the start of the QRS complex on the ECG. The atrial rheobase was unaltered (Fig. 4C), while the input current following PA‐6 to elicit action potentials in the ventricle was approximately doubled (Fig. 4D). To address the effect of PA‐6 on electrical stability of the atria and ventricle the duration and frequency of arrhythmia following S1–S2 stimulations were analyzed (Fig. 4E,F). The duration of arrhythmia in the atria was longer at 90 min compared to baseline, but no difference was observed between the PA‐6 group and the time‐matched control (DMSO) group (Fig. 4E). In contrast, ventricular arrhythmia lasting more than 1 sec was observed in two hearts in the PA‐6 group but in none of the time‐matched control (DMSO) hearts (Fig. 4F).

**Figure 5:**
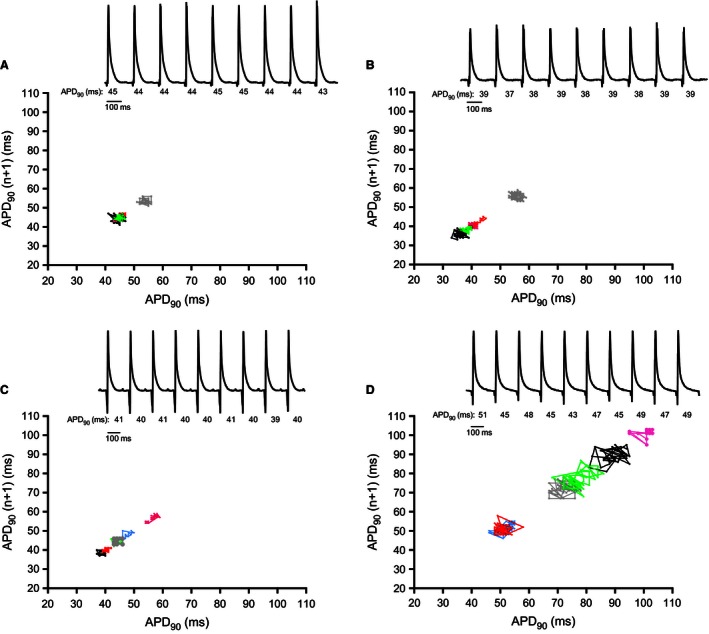
Ventricular beat‐to‐beat variability following PA‐6 infusion. Poincaré plots of 40 *n* + 1 ventricular APD_90_s. Baseline recordings performed before addition of 200 nmol/L PA‐6; (C) or equivalent amounts of DMSO (A). DMSO 90 min showed stable beat‐to‐beat variability over time (B). 200 nmol/L PA‐6 increased beat‐to‐beat variability in the rat ventricle (D). DMSO *n* = 5; PA‐6 *n* = 6. Representative MAP traces are shown to illustrate the beat‐to‐beat variability in APD_90_′s in msec, (A–D).

**Figure 6:**
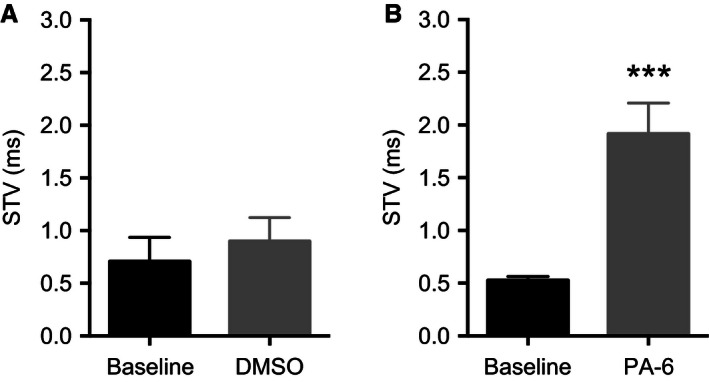
Short‐term variability in the ventricle. Data from right ventricle calculated from 40 subsequent APD_90_s. Two‐tailed paired t‐tests were used to compare baseline with the effect of 200 nmol/L PA‐6. (A) No effect of DMSO after 90 min (*n* = 5, ns, *P* = 0.5). (B) 200 nmol/L PA‐6 increased the short‐term variability (*n* = 6, *P* = 0.0008), *P < 0.05 was considered significant. **P < 0.01, ***P < 0.0001).

To investigate beat‐to‐beat variability in the ventricles Poincaré plots were made by plotting the current APD_90_ value against the preceding APD_90_ value. In the DMSO group the beat‐to‐beat variability was not changed after application of DMSO compared to baseline, *t* = 0 and at *t* = 90 (Fig. 5A,B), as quantified by calculating short‐term variability (STV) (Fig. 6). Application of PA‐6 resulted in a prominent change in beat‐to beat variability, as illustrated in the monophasic action potential recordings with indications of the individual APD_90_ values (Fig. 5D), Consequently, short‐term variability (STV) showed a significant difference after application of 200 nmol/L PA‐6 (*P* < 0.001, *n* = 6) (Fig. 6B).

## Discussion

At this point, only few chemical entities blocking the important cardiac K_ir_2.1 have a convincingly resolved mode of action. Recently pentamidine was used as a lead compound in the development of new potent and selective *I*_K1_ blockers (Takanari et al. 2013). 200 nmol/L PA‐6 blocked more than 90% of all K_ir_2.x channels in a voltage independent manner, yielding IC_50_ values of 12–15 nmol/L.

This study investigates the effect of PA‐6 on *I*_K1_ in an intact heart (Langendorff) preparation. The onset of PA‐6 mediated effects showed a time‐latency in the Langendorff‐preparations, reaching a plateau after 90 min. The slow onset of effect is probably due to PA‐6 blocking the channel pore from the cytosolic side as reported by De Boer et al. who applied the pentamidine compound from either the extracellular or the intracellular side of the membrane using the whole‐cell or inside‐out patch‐clamp technique (de Boer et al. 2010b). When testing the time‐dependency of PA‐6 block on HEK‐293/Kir2.1 cells by whole‐cell patch clamping we also observed a profound time‐dependency of drug effect, which manifested in a 20 min latency before 200 nmol/L PA‐6 produced its maximal block of the *I*_K1_ current (Fig. 1B).

Pharmacological modulation of *I*_K1_ has previously been reported to impact the electrical stability of the heart (Jalife and Berenfeld 2004). Suppression of *I*_K1_ increases APD/QT and depolarizes the diastolic potential, which may result in both early and delayed after‐depolarizations, while enhancing *I*_K1_ shortens APD/QT (Dhamoon and Jalife 2005). In agreement with this, we found that inhibition of *I*_K1_ in the ventricles using 200 nmol/L PA‐6 prolonged APD_90_ and increased vERP. In addition, we found that *I*_K1_ inhibition increased the variability in the ventricular action potential duration. This may be arrhythmogenic due to the proarrhythmic effect of enhanced dispersion/heterogeneity in ventricular conduction and repolarization (Thomsen et al. 2004; Szentandrassy et al. 2015). Variation in repolarization was determined by analyzing the beat‐to‐beat variability in APD_90_. Poincaré plots revealed that PA‐6 significantly increased beat‐to‐beat variability and STV in this isolated heart model. The variability in action potential duration found by Takanari et al. in canine ventricular myocytes Takanari et al. 2013) thereby also seems to translate to the whole beating heart. During the 90 min perfusion two of the six PA‐6 challenged hearts developed runs of ventricular arrhythmia following S_2_ stimuli, suggesting that *I*_K1_ inhibition increases the risk of ventricular arrhythmia.

The atrial‐ventricular conduction time was also increased by *I*_K1_ inhibition and because the Wenckebach cycle length was not changed this indicates a conduction slowing in the myocardium and not of the AV nodal tissue. As *I*_K1_ is known to play a pivotal role in setting the ventricular resting membrane potential (Dhamoon and Jalife 2005) it is expected that PA‐6 block induces a depolarization of the resting membrane potential, which will increase the fraction of inactivated cardiac sodium channels and thus lowered tissue excitability. Furthermore, a reduced availability of sodium channels will slow conduction due to the increased source‐to‐sink mismatch (Comtois et al. 2005). The increased risk of ventricular arrhythmia observed following loss of *I*_K1_ current (reviewed in [Dhamoon and Jalife 2005]) may thereby be supported by at least three different parameters being (1) APD_90_ dispersion; (2) prolonged vERP, and (3) reduced sodium channel availability caused by a depolarized RMP.

In the atria *I*_K1_ is believed to play a central role, but other repolarizing potassium currents, including *I*_K,ACh_ and *I*_K,Ca_ are also important in the later part of the action potential and in setting the resting membrane potential (Gaumond and Fried 1986; Wang et al. 2013; Skibsbye et al. 2014; Tang et al. 2015; van der Heyden and Jespersen 2016). Surprisingly, application of PA‐6 did not significantly alter aERP or the duration of the S_2_‐induced runs of atrial arrhythmia. In rabbit and human *I*_K1_ has been reported to be 6‐ to 10‐fold less expressed in atria as compared to ventricle (Giles and Imaizumi 1988; Wang et al. 1998). A similar lower atrial *I*_K1_ in the rat myocardium could explain the lack of PA‐6 effect in rat atria. It could also be speculated that other K^+^ currents, constituting an atrial repolarization‐ and resting membrane potential reserve, would ensure electrical stability even when *I*_K1_ is blocked.

For many years studies of the electrophysiological function of *I*_K1_ has been limited because of the lack of selective tool compounds. However, a selective inhibitor may not only be interesting as a tool for understanding cardiac *I*_K1_ properties in vitro it might also have therapeutic potential. The genesis and progression of atrial fibrillation has been suggested to be linked to an increased *I*_K1_ (Bosch et al. 1999; Ehrlich 2008). Likewise the upregulation of *KCNJ2/*Kir2.1 expression governed by changes in miR‐26 transcriptional levels has been linked to the promotion of clinical AF (Luo et al. 2013). It can therefore be suggested that selective pharmacological inhibition of *I*_K1_ could serve as an antiarrhythmic principle. However, the risk of ventricular side effects would limit clinical application. Further, short‐QT syndrome is associated with ventricular arrhythmias and sudden cardiac death (Pattnaik et al. 2012), and gain of function mutations in *KCNJ2* may result in arrhythmias related to Short‐QT syndrome (El Harchi et al. 2008; Hattori et al. 2012). Selective pharmacological inhibiting of *I*_K1_ might be useful in treating patients carrying such channelopathies, although the pharmaceutical interest might be limited due to the low number of patients diagnosed with such phenotypes worldwide.

### Study limitations

*I*_K1_ has a prominent role in controlling the resting membrane potential. Therefore, we attempted to test PA‐6's effect on rat atrial and ventricular tissue strips using microelectrode recordings to measure action potentials (data not shown). However, superfusion of PA‐6 had no effect on the electrophysiological properties of neither isolated atrial nor ventricular tissue, possibly due to low tissue penetrance of the drug as permeability is indeed reduced in a superfused tissue strip as compared to a coronary‐perfused Langendorff heart. Such permeability issues were previously observed for other compounds (Skibsbye et al. 2014). Also, experiments were performed in rat hearts which have different spatial and temporal distribution of ionic currents compared to larger mammals and humans. Hence, translatability of these findings to larger animals or humans should be done with caution. Exploring the ability of the PA‐6 compound to influence cardiac electrophysiology in larger animals is therefore needed.

## Conclusion

PA‐6 inhibits cardiac *I*_K1_ in Langendorff‐perfused rat hearts, resulting in prolonged ventricular action potentials and increased refractoriness. Moreover, block of *I*_K1_ in the rat ventricles also increases beat‐to‐beat variability and STV, as well as the risk of electrical stimulated ventricular arrhythmias. Atrial electrophysiology was not altered following *I*_K1_ block. These results suggest that blocking *I*_K1_ in rat hearts is proarrhythmic and may lead to increased susceptibility to ventricular arrhythmia.

## Conflict of Interest

None declared.
